# The Associations of Breastfeeding and Postnatal Experiences With Postpartum Depression Among Mothers of Hospitalized Infants in Tertiary Hospitals

**DOI:** 10.7759/cureus.29425

**Published:** 2022-09-21

**Authors:** Nurul Husna Mohd Shukri, Olivia Senjaya, Zurina Zainudin, Maslina Mohamed, Farah Inaz Syed Abdullah

**Affiliations:** 1 Department of Nutrition, Faculty of Medicine and Health Sciences, Universiti Putra Malaysia, Serdang, MYS; 2 Institute for Social Science Studies, Universiti Putra Malaysia, Serdang, MYS; 3 Department of Dietetics, Faculty of Medicine and Health Sciences, Universiti Putra Malaysia, Serdang, MYS; 4 Department of Paediatrics, Faculty of Medicine and Health Sciences, Universiti Putra Malaysia, Serdang, MYS; 5 Department of Paediatrics, Hospital Putrajaya, Putrajaya, MYS; 6 Department of Paediatrics, Hospital Tunku Azizah, Kuala Lumpur, MYS

**Keywords:** malaysia, mental health, perinatal mental health, hospitalized baby, lactation, maternity care, pediatric care, maternal distress, baby care, lactating mothers

## Abstract

Background

Postpartum depression has been linked to undesirable outcomes for mother-infant dyads, interfering with childcare and breastfeeding practices. This study aimed to determine the prevalence of depressive symptoms among mothers during the postpartum period and its association with breastfeeding and postpartum experiences.

Methodology

This cross-sectional study involved mothers of hospitalized infants (n = 219) at two tertiary hospitals in Klang Valley, Malaysia. Mothers were screened for postpartum depression using the Edinburgh Postnatal Depression Scale with a cut-off of ≥12 for positive screening for depression. Mothers were asked to complete questionnaires on breastfeeding experience, which included breastfeeding self-efficacy and challenges. The questionnaires also collected information on postnatal experiences, including birth outcomes, anxiety and stress levels, and social support. Multiple linear regression was used to ascertain the association of postpartum depression levels with breastfeeding and postnatal experiences.

Results

Overall, 30% of mothers in this study screened positive for depression. Based on multiple linear regression, a higher score of postpartum depression was significantly associated with unpleasant breastfeeding and postnatal experiences reflected by increased scores of anxiety and stress, lower infant birth weight, increased breastfeeding problems, and lower level of social support (p < 0.005).

Conclusions

Maternal emotions, birth outcomes, breastfeeding issues, and social support were associated with postpartum depression. Efforts should be made to increase maternal support, and screening for maternal depression during infant hospital stays should be encouraged.

## Introduction

Maternal mental health is one of the potential factors that can have a significant impact on a child’s well-being. Postpartum depression is a type of depression that occurs in women after giving birth, with symptoms such as distressing mood, severe anger, difficulty sleeping, and frequent mood changes. Postpartum depression affects the health, relationships, and quality of life of mothers, which, in turn, results in a low-quality home environment and reduced maternal sensitivity in childcare, negatively affecting child development [1].

Surveillance data from the Centers for Disease Control and Prevention (CDC) indicate that one in eight mothers experience symptoms of postpartum depression after birth [2]. The prevalence of postpartum depression in Asian countries ranged from 3.5% to 63.3% [3], while a review reported that postpartum depression among Malaysian mothers is 17.05% [4]. Hence, there is an urgent need to identify risk factors for postpartum depression and efforts to reduce psychological distress and promote the well-being of mother and child, particularly during infant hospitalization, when the rate of postpartum depression is higher [5].

Breastfeeding has long been recognized as the gold standard for infant care, which provides health benefits to both the mother and the infant. Breastfeeding is associated with a reduced risk of breast cancer, ovarian cancer, and type 2 diabetes in mothers, as well as a reduced risk of acute illness and gastrointestinal problems in infants [6,7]. It has been shown that breastfeeding has long-term benefits that lower the rate of obesity and predict better health and cognitive development later in life [7].

However, postpartum maternal psychological distress seems to be an obstacle to achieving successful breastfeeding. A systematic review found an association between breastfeeding duration and maternal depression, although it was unclear which was the major reason; either being depressed shortened the duration of breastfeeding, or early discontinuation of breastfeeding increased depressive symptoms [8]. Nevertheless, prenatal distress was also reported to lead to an early cessation of breastfeeding [9]. A recent longitudinal study reported that exclusive breastfeeding reduces the link between prenatal and postpartum depression. At three months postpartum, mothers whose infants were exclusively breastfed were less likely to be depressed than mothers whose infants had started to be given infant formula [10]. Hence, it is critical to identify the maternal psychological state during the early postpartum period, and its association with breastfeeding and other maternity experiences, especially among mothers of higher-risk groups.

To date, there are few studies on the prevalence of psychological distress among mothers of hospitalized infants, specifically in Malaysia, as the majority of mental health studies focus on the general population [4]. Thus, it is important to explore postpartum depression in the hospital setting to better understand the current maternal situation and what can be done to improve the psychological and physical outcomes of both mothers and infants. Moreover, because mothers of hospitalized infants are more sensitive and vulnerable during the postpartum period, determining the prevalence of postpartum depressive symptoms among them in Malaysia is crucial.

Therefore, this study aimed to identify the characteristics of mothers suffering from depressive symptoms or target groups, as well as modifiable factors for future interventions. The purpose of this study was to assess the prevalence of depressive symptoms among mothers of hospitalized infants in tertiary hospitals, as well as its association with breastfeeding and postpartum experiences.

## Materials and methods

Study design

A cross-sectional study was conducted among mothers of hospitalized infants in two general hospitals in Klang Valley, Malaysia. Ethical approval to conduct the study was granted by the Medical Research and Ethics Committee (MREC) Malaysia in January 2020 (ID: NMRR-19-3216-51651). All participants provided written informed consent forms upon participation in the study.

Study population

Malaysian mothers aged 19 to 45 years old with infants (aged seven months and under) requiring pediatric treatment or care (e.g., hospitalized for medical treatment or followed-up at pediatric clinics for a previous hospitalization) were eligible to participate in the study. Mothers who did not speak English or Malay, had never breastfed, were on medication for any disease(s), had a history of mental illness, or smoked were excluded from the study. To increase the homogeneity of the study population, these inclusion criteria targeted healthy mothers. Based on the regression sample size formula, a minimum sample size of 210 mothers (adjusted for an 80% response rate) was required for this study. Using a purposive sampling method, 331 mothers were approached to participate in the study, of whom 294 met the inclusion criteria and 219 agreed to participate and completed the questionnaires.

Data collection

Data collection was carried out between January 1st and March 1st, 2020. Mothers were approached, screened, and recruited at the pediatric wards, special care nursery (SCN), neonatal intensive care unit (NICU), and outpatient pediatric clinics. For each mother, an information sheet was provided, and written consent was obtained from all respondents who agreed to participate in the study. During data collection, all participants were screened for depression and assessed for stress and anxiety levels. They also completed a questionnaire on obstetrical characteristics, social support, and breastfeeding experiences, including self-efficacy and challenges. The infant’s medical data were obtained from the health record book.

Postpartum depression

Maternal postpartum depression levels were measured using the Malay and English versions of the Edinburgh Postnatal Depression Scale (EPDS) [11]. The EPDS has been widely used as a screening tool for postpartum depression and has been tested for reliability in many previous studies, including in Malaysia, with a strong internal consistency of α ≥ 0.84 [12]. The EPDS consists of 10 items with a total score ranging from 0 to 30. A score of 12 or higher was used as the cut-off point for positive screening of depression because it has been shown to have the highest sensitivity and specificity for the Malay version of EPDS [13]. The calculated Cronbach’s alpha for the EPDS of this study was 0.80.

Maternal and infant characteristics

The sociodemographic data regarding maternal age, ethnicity, marital status, education level, occupation, and household income were obtained. Information on the infant’s sex, age, gestational week at birth, birth weight, and weight-for-age (WAZ) were obtained from the infant’s health record at the hospital. WAZ is further classified according to the WHO Child Growth Standard 2006.

Breastfeeding experience

Breastfeeding experience encompasses breastfeeding self-efficacy and challenges. Breastfeeding self-efficacy is defined as maternal confidence in the ability to breastfeed their infant. Breastfeeding self-efficacy was assessed using the Breastfeeding Self-Efficacy Scale - Short Form (BSES-SF) [14]. The questionnaire consists of 14 items with a five-point scale ranging from “not at all confident” to “very confident.” The maximum score of BSES-SF is 70, with higher self-efficacy when the score is high. Both the English and Malay versions of BSES-SF have been validated and found to be reliable (α ≥ 0.90) [15], with a high calculated internal consistency of Cronbach’s alpha (0.96) in this study. An interquartile range (IQR) was used to further categorize the score of breastfeeding self-efficacy into low, moderate, and high. Breastfeeding challenges (i.e., pain and problems) were assessed using a questionnaire adapted from the Infant Feeding Practice Study II (IFPS II) Neonatal Questionnaire by the CDC [16]. The Malay version of the adapted questions has been used previously among Malaysian mothers [12].

Postnatal experiences

Postnatal experience encompasses the assessment of maternal anxiety and stress levels during the postpartum period, support received after birth, and obstetric outcomes.

Maternal anxiety was assessed using the Beck Anxiety Inventory (BAI), which consists of 21 items rated on a four-point Likert scale. BAI scores ranged from 0 to 63, with a suggested clinical cut-off point of 16 [17]. The higher the score, the greater the likelihood of the respondent experiencing anxiety. Both the English and Malay versions of BAI have been validated and found to be reliable (α ≥ 0.91) [18]. Consistently, the calculated Cronbach’s alpha of BAI in this study was 0.95. The Perceived Stress Scale (PSS) was used to assess the degree to which mothers perceive their lives as stressful [19]. The Cronbach’s alpha of PSS in this study was 0.74, which is considered reliable. PSS consists of 10 items, and the scoring is obtained by summing up the total score (reversing response for items 4, 5, 7, and 8, for which 0 = 4, 1 = 3, 2 = 2, 3 = 1, and 4 = 0) [19]. The scores ranged from 0 to 40, with higher scores indicating a higher level of stress.

Obstetric outcomes and birth experiences were assessed through a self-administered questionnaire, adapted from the Neonatal Questionnaire of Infant Feeding Practices Study II [16]. Attributes in this section were parity, pregnancy weight gain, family planning, delivery method, skin-to-skin contact, and breastfeeding initiation, and were available in both Malay and English versions. This adapted questionnaire has been used previously among the Malaysian population [12].

Social support during the postpartum period was assessed through a self-administered questionnaire of the Multidimensional Scale of Perceived Social Support (MSPSS). MSPSS is a valid and reliable tool to assess perceived social support from friends, family, and significant others with an internal consistency of α = 0.91 [20]. It consists of 12 items with seven possible responses to each statement (scoring ranged from 0 to 6 with a maximum score of 72). A higher score indicates higher perceived social support. The Malay version of MSPSS has been validated among the Malaysian population with an overall Cronbach alpha of 0.89, whereas the calculated Cronbach’s alpha was found to be higher in this study (0.93). We used the IQR to divide the social support score into three categories, namely, low, moderate, and high.

Statistical analysis

Data were analyzed using SPSS version 25 with a statistical significance of p < 0.05. Normality was assessed for continuous data. Means and standard deviation (SD) were presented for normally distributed data, while median and IQR were presented for non-normally distributed data. Multiple linear regression analysis was performed using the stepwise method to seek associations of maternal-infant factors with postpartum depression scores. Sociodemographic variables were included in the model to adjust for potential confounders.

## Results

The psychological state of mothers in the postpartum period

Table 1 shows that the prevalence of positive postpartum depression in this study based on depressive symptoms of EPDS screening was 30.1% (n = 66), with another 12.8% at risk of possible depression. In addition, one-fourth of the mothers experienced clinically significant anxiety, as indicated by the BAI score of 16 or above. Meanwhile, the mean score of stress was 16.7 ± 5.4, showing that mothers in this study had a moderate level of postpartum stress.

**Table 1:** Maternal psychological state of the study participants (n = 219). ^a^: The Edinburgh Postnatal Depression Scale (EPDS) score of 12 or above (out of 30) is considered the clinical cut-off point for positive screening of depression. ^b^: A Beck Anxiety Inventory (BAI) score of 16 or above is considered the clinical cut-off point for anxiety. ^c^: The Perceived Stress Scale (PSS) has a maximum score of 40. A higher score indicates a higher level of stress.

Maternal psychological state	n	%	Mean	SD	Median	IQR
EPDS score (depression)^a^			9.4	5.3		
Not likely	125	57.1				
Possible depression	28	12.8				
Positive screening	66	30.1				
BAI score (anxiety)^b^					8.0	3.0, 16
Clinically insignificant	164	74.9				
Clinically significant	55	25.1				
PSS score (stress)^c^			16.7	5.4		

Maternal sociodemographic characteristics

Table 2 shows the sociodemographic characteristics of the study population. The mean age of mothers was 31.6 ± 4.9 years, with the majority being Malay (90.5%), having tertiary education (38.4%), employed (75.3%), and having a low household income (46.7%).

**Table 2:** Sociodemographic characteristics of the study participants (n = 219). Note. USD refers to the United States dollar while RM refers to Ringgit Malaysia. ^a^: USD 1 = RM 4.45 conversion rate in August 2022.

Characteristics	n	%	Mean	SD
Age (years)			31.6	4.9
Ethnicity				
Malay	198	90.5		
Chinese	11	5.0		
Indian	6	2.7		
Bumiputera	4	1.8		
Marital status				
Married	216	98.6		
Education level				
Primary/secondary education	57	26.0		
Pre-University	78	35.6		
Tertiary education	84	38.4		
Occupation				
Employed	165	75.3		
Unemployed	54	24.7		
Household income^a^				
Low (<3,800)	100	46.7		
Middle (RM 3,800–8,300)	82	38.3		
High (>RM 8,300)	32	15.0		

Obstetric and infant characteristics at birth

Table 3 shows that three out of ten mothers had their first child, and the majority had a vaginal delivery (64%). This study found that 80% of mother-infant dyads had skin-to-skin contact within the first hour of life, and 57.5% had skin-to-skin contact directly after birth. Nearly 60% of mothers attempted breastfeeding within the first hour of life. Among 219 infants, 58% were male, with the majority being born at full term (76.2%) and with a normal birth weight (77%). Medical conditions of infants in this study were being preterm (23.3%), having jaundice (33%), and other health conditions (20%), including pulmonary condition, heart condition, sepsis, bone disease, esophageal atresia, and others. The median age of infants was 24 days (IQR = 5, 90), with the majority being less than a month old (51.6%).

**Table 3:** Obstetric and infant characteristics (n = 219). Note: Infants in this study were hospitalized or being followed up at hospital clinics due to certain health conditions that required pediatric care. ^a^: Any conditions that apply to infants (it could be more than 1). ^b^: Other medical conditions at birth include pulmonary conditions, heart conditions, sepsis, bone disease, esophageal atresia, and others. *: Data presented as median (IQR).

Characteristics	n	%	Mean	SD
Obstetric characteristics				
Infant parity				
1	66	30.0		
≥2	153	70.0		
Method of delivery				
Vaginal (natural)	105	47.9		
Vaginal (induced)	36	16.4		
Cesarean delivery	78	35.6		
Skin-to-skin contact				
Directly after birth	126	57.5		
Within 60 minutes	49	22.4		
>60 minutes	44	20.1		
First breastfeeding attempt				
Directly after birth	54	24.7		
Within 60 minutes	75	34.2		
>60 minutes	90	41.1		
Infant characteristics				
Sex				
Male	127	58.0		
Female	92	42.0		
Age (Days)			52.4	57.2
<1 month	113	51.6		
1–4 months	71	32.4		
4–6 months	35	16.0		
Gestational week at birth			37.2	2.5
>37 weeks (full-term)	168	76.7		
<37 weeks (preterm)	51	23.3		
Birth weight (kg)			2.8	0.6
>2.5 kg	167	77.0		
<2.5 kg (low birth weight)	50	23.0		
Normal weight	84	39.1		
Underweight	22	10.2		
Severely underweight	109	50.7		
Medical conditions^a^				
Jaundice	72	33.0		
Fetal distress	14	6.4		
G6PD deficiency	11	5.0		
Others^b^	44	20.2		
Hospitalization (days)			5.6	13.4

Breastfeeding experience

Based on Table 4, the average score of breastfeeding self-efficacy was 56.72 (SD = 10.73), or 80% of the total score, indicating an acceptable level of confidence in breastfeeding during the postpartum period. However, when scores were categorized into low, moderate, and high levels of confidence, 37% of mothers were categorized as having low levels of breastfeeding self-efficacy. Breastfeeding pain and problems were found to be prevalent, with more than half reported having experienced pain while breastfeeding at any time during the postpartum period. Moreover, 39.3% of mothers experienced one to two breastfeeding problems, while the other 40% were found to encounter at least three problems. Specifically, at certain time points, the mean pain levels on the first day, first week, and second week were 3.6 (SD = 2.4), 3.7 (SD = 2.4), and 3.0 (SD = 2.3), respectively.

**Table 4:** Breastfeeding challenges, self-efficacy, and maternal social support (n = 219). ^¶^:Breastfeeding pain level, measured on a scale of 1-10. ^‡^: Breastfeeding self-efficacy was measured using the Breastfeeding Self-Efficacy Scale Short Form (BSES-SF) with a maximum score of 70. A higher score indicates higher self-efficacy. ^§^: Maternal support was measured using the Multidimensional Scale of Perceived Social Support (MSPSS) with a maximum score of 72. A higher score indicates more support from family, friends, and significant others.

Characteristics	n	%	Mean	SD
Number of breastfeeding problems				
0	46	21.0		
1–2	86	39.3		
3–4	48	21.9		
≥5	39	17.8		
Breastfeeding problems				
None	67	30.6		
Sore/cracked/bleeding nipple	73	33.3		
Baby had trouble sucking or latching	66	30.1		
Baby would not wake up	55	25.1		
I did not have enough milk	51	23.3		
Baby nursed too often	50	22.8		
Engorgement (breast too full)	48	21.9		
Took a long time for my milk to come in	37	16.9		
I had trouble initiating milk flow	32	14.6		
Baby did not get enough/lose weight	28	12.8		
Clogged milk duct	28	12.8		
Baby choked	23	10.5		
Breast leaked too much	21	9.6		
Baby got distracted/not interested	26	11.9		
Pain during breastfeeding	218	99.5		
Yes	121	55.5		
No	97	44.5		
Breastfeeding pain level^¶^				
First day			3.6	2.4
First week			3.7	2.4
Second week			3.0	2.3
Breastfeeding self-efficacy^‡^			56.7	10.7
Low	82	37.4		
Moderate	60	27.4		
High	77	35.2		
Social support score^§^			58.4	10.6
Low	74	33.8		
Moderate	81	37.0		
High	64	29.2		

Social support

The average score of MSPSS was 58.37/72 (SD = 10.62), indicating a relatively high social support received by the majority of mothers. However, further categorization into low, moderate, and high support showed that about one-third of the study population had low social support during the postpartum period.

Factors associated with postpartum depression

Variables that were found to be significantly correlated with postpartum depression in univariate analyses were then analyzed in a multiple linear regression to identify risk factors that predict the severity of postpartum depression. Household income, education level, postpartum anxiety, postpartum stress, social support, breastfeeding self-efficacy, infant parity, breastfeeding pain and problems, infant age, sex, gestational age at birth, and birth weight were included in the analysis. The final regression model shows that increased scores of anxiety and stress, lower infant birth weight, an increased number of breastfeeding problems, and lower social support levels were significantly associated with a higher score of postpartum depression (p < 0.05) (Table 5). Based on the adjusted R-square value, the independent variables in this study explained 55.5% of the variability of depression score levels.

**Table 5:** Predictors of postpartum depression analyzed using a stepwise regression method. Note: Edinburgh Postnatal Depression Scale (EPDS) score is the dependent variable. The statistical significance level (sig.) is set at p < 0.05. B: unstandardized beta; Std. Error: standard error; Sig.: significance/p-value; BAI: Beck Anxiety Inventory; PSS: Perceived Stress Scale; No. of BF problems: number of breastfeeding problems; MSPSS: Multidimensional Scale of Perceived Social Support *: Model 5 adjusted R-square value is 0.555.

		Unstandardized coefficients		Standardized coefficients			95% confidence interval for B	
Model		B	Std. Error	Beta	t	Sig.	Lower bound	Upper bound
1	(Constant)	5.839	0.425		13.726	<0.001	5.000	6.678
	BAI total score	0.311	0.026	0.650	11.924	0.000	0.259	0.362
2	(Constant)	0.397	0.860		0.462	0.645	-1.298	2.093
	BAI total score	0.219	0.027	0.458	8.198	<0.001	0.166	0.271
	PSS total score	0.390	0.055	0.394	7.059	<0.001	0.281	0.500
3	(Constant)	3.695	1.493		2.476	0.014	0.751	6.639
	BAI total score	0.221	0.026	0.462	8.404	<0.001	0.169	0.273
	PSS total score	0.377	0.055	0.381	6.904	<0.001	0.270	0.485
	Birth weight (kg)	-1.101	0.411	-0.129	-2.682	0.008	-1.911	-0.291
4	(Constant)	3.397	1.488		2.282	0.024	0.461	6.332
	BAI total score	0.213	0.026	0.446	8.072	<0.001	0.161	0.265
	PSS total score	0.366	0.055	0.369	6.701	<0.001	0.258	0.473
	Birth weight (kg)	-1.106	0.407	-0.130	-2.715	0.007	-1.909	-0.302
	No. of BF problems	0.246	0.122	0.099	2.012	0.046	0.005	0.487
5*	(Constant)	6.728	2.140		3.144	0.002	2.506	10.949
	BAI total score	0.209	0.026	0.438	7.999	<0.001	0.158	0.261
	PSS total score	0.342	0.055	0.346	6.207	<0.001	0.233	0.451
	Birth weight (kg)	-1.042	0.405	-0.122	-2.577	0.011	-1.841	-0.244
	No. of BF problems	0.281	0.122	0.113	2.301	0.022	0.040	0.522
	MSPSS total score	-0.054	0.025	-0.106	-2.148	0.033	-0.103	-0.004

## Discussion

Positive screening for maternal depressive symptoms was found to be prevalent in this study, with 30% of mothers screening positive for depression during the postpartum period. This is higher than the previous Malaysian studies, which reported a range from 6.8 to 27.3% [2,4]. In addition, about two to three in ten mothers were screened to have clinically significant postpartum anxiety in the study. Because the infants in this study had medical issues that required specialized hospital care, it was presumed that their mothers were more likely to experience emotional distress. This study also found that postpartum anxiety and stress, infant birth weight, the number of breastfeeding problems, and social support were significant factors associated with postpartum depression.

It is well-established that social support counters the undesirable effects of stressful life events and protects against postpartum depression [21]. A review reported that depressed mothers felt isolated, especially if they were experiencing a lack of support from their partners and health professionals after discharge [22]. Hence, additional support from family, friends, and significant others plays an important role in reducing postpartum depression and increasing maternal self-efficacy through a sense of belonging, companionship, and infant care [21]. The finding of this study agrees with the literature, which suggests that social support negatively predicts postpartum depression, as shown in the final regression model. Therefore, identifying the high-risk group of mothers is critical in assisting them in reaching out for help or seeking support.

Low birth weight, which usually relates to gestational age, is one of the risk factors for postpartum depression [23]. Although this study did not find a significant relationship between gestational age and postpartum depression, birth weight has been found to be a significant predictor of postpartum depression. A recent clinical intervention found that staff training, with emphasis on communicating infant behavior and needs, active listening to parents’ perception, collaborative planning and involvement of infant care, and decision-making with parents, is effective in reducing the symptoms of postpartum depression in mothers with very low-birth-weight (preterm) infants [24]. Hence, future interventions should target more vulnerable mothers, specifically mothers of low-birth-weight or preterm infants.

During the postpartum period, mothers who had low breastfeeding self-efficacy as well as breastfeeding problems or difficulties, such as pain, discomfort, and concern, were more likely to develop depressive symptoms [8]. Nevertheless, it has been suggested that mothers will adapt and develop a coping mechanism that will result in fewer breastfeeding problems [25]. Breastfeeding self-efficacy was found to be inversely correlated with postpartum depression (p < 0.001), although it was not found to be a significant predictor of postpartum depression in the final regression model of this study. In contrast with this finding, other studies reported that breastfeeding self-efficacy significantly predicts mood outcomes, including postpartum depression [15,26]. Postpartum depression has also been shown to significantly predict breastfeeding duration and exclusivity [15]. Hence, improved self-efficacy through educational and emotional support may lead to reduced depression and improved breastfeeding practice and exclusivity, which have been widely shown to improve maternal and infant health and outcomes.

Consistent with previous studies, stressful life events, including anxiety, are significant predictors of postpartum depression [27]. The coronavirus disease 2019 (COVID-19) pandemic is one example of a stressful life event that can potentially increase the levels of stress and anxiety due to unemployment, financial difficulties, loss of loved ones, and fear or stress of contracting the virus, including in Malaysia [28]. A recent review indicated there was a tremendous increase in postpartum depression and anxiety among mothers of infants or young children during the COVID-19 pandemic [29]. Consequently, while postpartum women are in a more vulnerable state, it is vital to screen them for mental health conditions and provide the necessary support and intervention. This is especially critical for high-risk women, namely, those whose infants require specialized care or were born prematurely.

In addition to screening, efforts need to be made to reduce the prevalence of postpartum depression, including active referrals to relevant healthcare professionals for mothers at risk or who screen positive for postpartum depression. Additionally, staff training [24] and relaxation interventions for mothers [30] may be implemented as both interventions have shown great potential in reducing maternal distress. More trials may be conducted in hospital settings to further assess the effects of both interventions among the Malaysian population.

Limitations

Despite the significant findings, there are several limitations to this study. The majority of mothers in this study were Malays, leading to a homogeneous study population. Thus, the results may not fully represent the multi-racial Malaysian population and cannot be generalized to the entire Malaysian population. In addition, this study was unable to establish a causal relationship due to its cross-sectional study design. For future studies, a larger sample size and the use of stratified random sampling to represent diverse ethnicities are recommended, as this may also alleviate bias in the recruitment process. A longitudinal study is also suggested to allow the progression of maternal mental health to be tracked and observed. Through a longitudinal study, different levels of maternal distress during different stages of the peripartum period and its associated factors may be observed. Hence, future assessments and interventions can be more precise, timely, and effective.

## Conclusions

Positive screening for postpartum depression is prevalent among mothers with infants requiring pediatric care in tertiary hospitals. Screening for maternal depression during infant hospital stays should be encouraged to identify mothers at risk of postpartum depression. Maternal anxiety and stress, infant birth weight, the number of breastfeeding problems, as well as social support were significant predictors of postpartum depression in this study.

Efforts are needed to reduce postpartum anxiety and postpartum stress, as well as improve the breastfeeding experience and social support. This includes active referrals to relevant healthcare professionals for mothers at risk or who screened positive for postpartum depression, increase health professional staff training, helping mothers to identify additional support from the community, and practicing relaxation interventions during the postpartum period. Consequently, this could help to reduce the incidence of postpartum depression as well as improve breastfeeding self-efficacy and duration. Close monitoring should also be done for more vulnerable mothers, especially those whose infants require pediatric care or were born prematurely.
